# The Novel Analogue of Hirsutine as an Anti-Hypertension and Vasodilatary Agent Both In Vitro and In Vivo

**DOI:** 10.1371/journal.pone.0119477

**Published:** 2015-04-24

**Authors:** Kai Zhu, Su-Na Yang, Fen-Fen Ma, Xian-Feng Gu, Yi-Chun Zhu, Yi-Zhun Zhu

**Affiliations:** 1 Department of Pharmacology, School of Pharmacy and Institute of Biomedical Sciences, Fudan University, Shanghai, 201203, China; 2 Department of Medicinal Chemistry, School of Pharmacy and Institute Biomedical Sciences, Fudan University, Shanghai, 201203, China; 3 Departments of Physiology and Pathophysiology, Shanghai College of Medicine, Fudan University, Shanghai, 201203, China; 4 Department of Pharmacology, National University of Singapore, Singapore, Singapore; Max-Delbrück Center for Molecular Medicine (MDC), GERMANY

## Abstract

In this paper, an analogue of hirsutine (compound **1**) has been synthesized and evaluated as an anti-hypertension agent, which exhibits extraordinary effects on the contractile response of thoracic aorta rings from male SD rats in vitro (IC_50_ = 1.129×10^-9^±0.5025) and the abilities of reducing the systolic blood pressure (SBP) and heart rate (HR) of SHR in vivo. The mechanism investigation reveals that the vasodilatation induced by compound **1** is mediated by both endothelium-dependent and -independent manners. The relaxation in endothelium-intact aortic rings induced by compound **1** can be inhibited by L-NAME (1×10^-6^ mol•L^-1^) and ODQ (1×10^-6^ mol•L^-1^). Moreover, compound **1** can also block Ca^2+^ influx through L-type Ca^2+^ channels and inhibit intracellular Ca^2+^ release while no effect on K^+^ channel has been observed. All these data demonstrated that the NO/cyclic GMP pathway can be involved in endothelium-dependent manner induced by compound **1**. Meanwhile the mechanism on the vasodilatation of compound **1** probably also related to blockade of Ca^2+^ influx through L-type Ca^2+^ channels and inhibition of intracellular Ca^2+^ release may have no relationship with K^+^ channels.

## Introduction

Hypertension has been considered as a common risk factor for progression of cardiovascular disorders and often accompanied by endothelium dysfunction, metabolic syndrome, diabetes, renal dysfunction, increased peripheral vascular resistance, and excessive activation of the renin-angiotensin system[1]. Each type of anti-hypertension medicine has its advantages and disadvantages, and that most patients with hypertension required life-long medication, the discovery of new agents with higher efficacy and lower toxicity has become one of the prime tasks for medicinal chemists and biologists[2].

*Uncaria rhynchophylla*, a traditional Chinese herb medicine, has been proven that it has a significant effect of anti-hypertension. Several integrant of *Uncaria rhynchophylla* has been isolated, such as rhynchophylla, hirsutine, corynantheine, isocorynoxeine, and all of them have been identified as indole alkaloids[3]. Recently, we reported that hirsutine, an integrant of *Uncaria Rhynchophylla*, has cardioprotection on hypoxic neonatal rat cardiomyocytes[4]. In order to acquire a deeper understanding and more detailed knowledge on the pharmacological functions and clinical applications of the indole alkaloids from *Uncaria rhynchophylla*, hirsutine and its analogues have been synthesized, and we find that compound **1**, one of analogues of hirsutine, exhibits remarkable antihypertensive and dilating effect both in vitro and in vivo. In the further studies, we also reveal that its dilation mechanisms are involved in both endothelium-dependent and—independent pathways, especially for the pathways modulated by NO and Ca^2+^ related proteins. In additionally, we observed that neither the non-specific ATP-sensitive K^+^ channel nor the Ca^2+^-activated K^+^ channel might have relationship with the vasodilation effect induced by compound **1**. With these results we has originally show the anti-hypertension effect and vasodilation mechanisms of compound **1** which are very important for it as a new anti-hypertension drug candidate.

## Materials and Methods

### Chemicals and drugs

**Fig 1:**
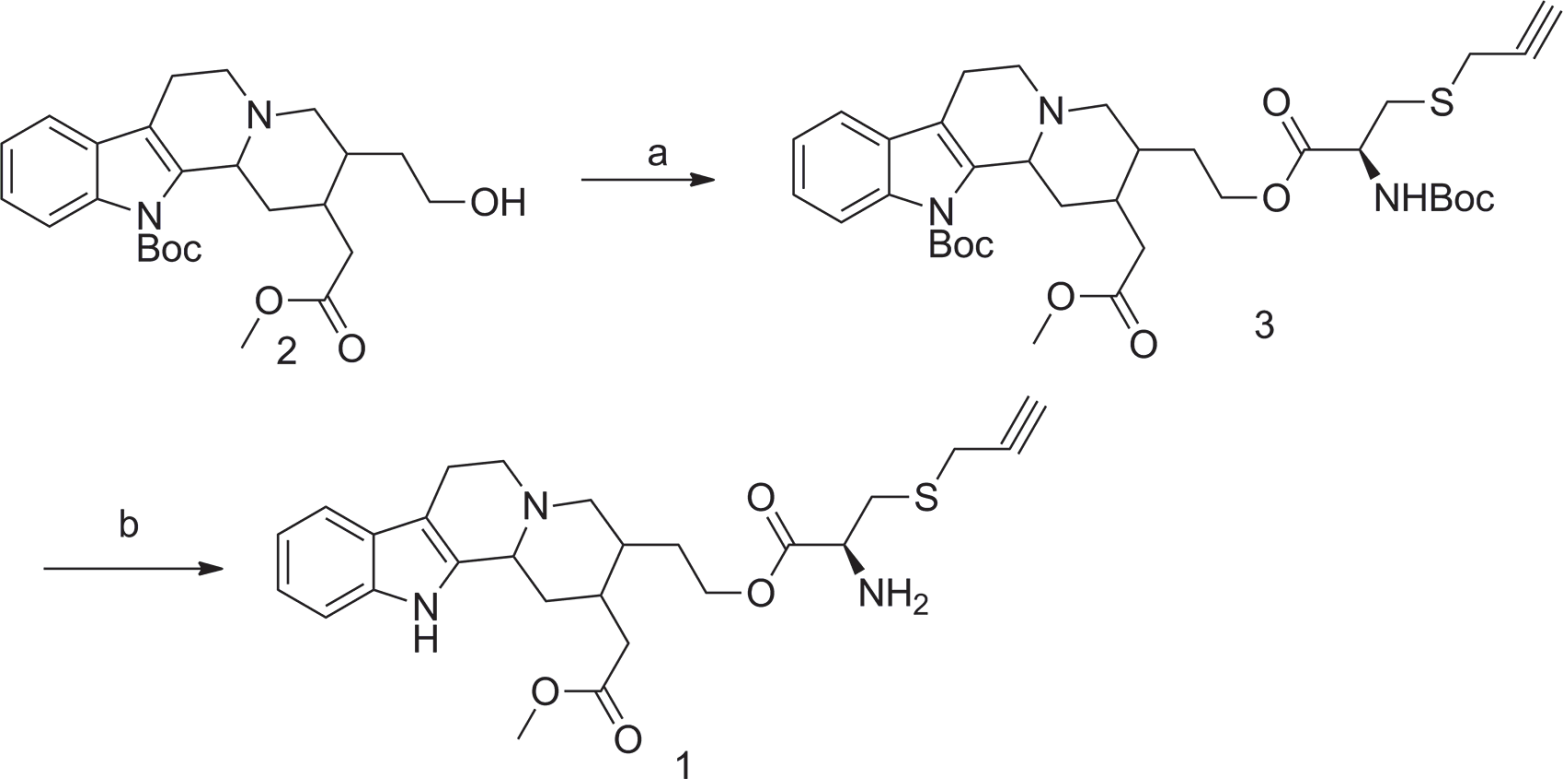
The synthetic route of compound 1.

Compounds **1** was prepared as outlined in Scheme 1. First of all, compound **2** was synthesized according to the method provided by literature[5]. And then, the condensation between compound **2** and (R)-2-((tert-butoxycarbonyl)amino)-3-(prop-2-yn-1-ylthio) propanoic acid was performed in the presence of DIC and DPTS to give compound **3** in 81% yield. Finally, the Boc group in compound **3** was removed under acidic condition, affording target compound **1** in 59% yield (**Fig 1)**.

N^G^-Nitro-L-arginine Methyl Ester (L-NAME), glibenclamide, indomethacin, nifedipine, TEA, captopril, atropine and ODQ were purchased from Sigma-Aldrich Co. (St. Louis, MO, USA). Phenylephrine (PE) and norepinephrine·HCl (NE) were obtain from Shanghai Hefeng Pharmaceutical Co.(China). All other reagents were analytical grade.

Compound **1** could be dissolved by distilled water and anhydrous ethanol and another reagents were stored as aqueous solution except glibenclamide and nifedipine, which must be dissolved in DMSO. The pre-study revealed that the dosage of DMSO and anhydrous ethanol had no influence on increasing isolated aorta tension or reducing blood pressure. All reagents should be pre-sterilizated during next experiments.

### Toxicity test by MTT and LDH Elisa kit

Firstly, cultured rat aortic smooth muscle cells as previously described[6]. Briefly, male SD rat (150–200 g) was anaesthetized using chloral hydrate (2mg/100g). The main aortic artery was dissected out and kept in ice-cold PBS with pH adjusted to 7.4 with NaOH. The artery was cut into 1mm^2^ pieces, and incubated in Collagenase II containing Penicillin and streptomycin at 37°C for 3 hours, then centrifuged the solution and removed the supernatant. Finally, place the single piece respectively on the petri dish and put into a little DMEM containing 20% supplemented fetal bovine serum (FBS) for a few days to let the VSMCs climb out from the issue.

The VSMCs were cultured in DMEM containing 10% FBS, 100 U/mL penicillin, and 100mg/mL streptomycin. The VSMCs were identified by SMC-specific α-actin monoclonal antibody (Sigma, Oakville, Ontario, Canada) which showed the obvious brown grain and the typical ‘hill-and-valley’ pattern[7]. Once the cells got 70–80% confluence, the experiments could be implemented, between passages 3 and 10.

Cell toxicity test was accessed by MTT (Sigma, St. Louis. MO, USA).Briefly, cells were plated on 96-well plates (approximately 6000 cells/well) in culture medium and maintained in regular growth medium for 2 days. Cells were pretreated for 12 h with compound **1** (1.6, 8, 40,200,1000μΜ), removed the medium and washed twice with phosphate-buffered saline (PBS). Incubated with MTT (500ug/mL) for 4h and add dimethylsulfoxide (150μl/well). After 10 min at room temperature, the absorbance was read at 490 nm, with 690 nm as reference. Cell viability (%) was calculated as the absorbance of each group divided by the absorbance of the normal control multiplied by 100[4].

Additional assessment of cell viability was conducted by measurement of LDH leakage from cells into the medium[8]. LDH (lactate dehydrogenase) released into the culture medium was determined using an LDH Assay Kit (Biotime, Haimen, China). Results were analyzed spectrophotometrically at 490 nm. Relative LDH release is expressed as a percentage of that of normal cells. Control (100%) was defined as LDH leakage into the medium from cells in normal samples.

### Effect of compound 1 in vasodilation and anti-hypertension

#### Vasodilation Activity

To determine the vasodilation activity of compound **1**, Sprague-Dawley rats (200–250g) were sacrificed after anesthesia with chloral hydrate (20mg/kg) and immerse the thoracic aortas in Physiological Salt Solution (PSS) which contains (mM): NaCl, 120.6; KCl, 5.9; CaCl_2_, 2.5; MgSO_4_, 1.2; KH_2_PO_4_, 1.2; NaHCO_3_, 15.4; glucose, 11.5, pH 7.4. Each aortas must be removed from tissue and fat, then have it cut into 3–4 rings (3–5 mm). A stainless steel triangular hook get each ring through and put them into a bath filled with 10ml PSS at 37°C with mixed gas of 95% O_2_ and 5% CO_2_. Then connect the rings with the tension transducer (Shanghai Jialong Instrument Factory, China) which has already connected to SUMP-PC biological signal processor (Shanghai Jialong Instrument Factory, China) so as to measure isometric tension in the vessels, under an optimal tension of 2g. The bath fluid was changed every 15 min.

The aortic rings with endothelium were pre-contracted with PE (10^–6^ mol·L^-1^). Adding cumulative compound 1 with the concentration gradually increasing from 10^–11^ to 10^–4^ mol·L^-1^ when the maximum tension was achieved to obtain the concentration–response curve of compound 1-induced relaxation. Endothelial integrity was identified by the level of relaxation caused by acetylcholine (10^–5^ mol·L^-1^) under the isometric tension induced by phenylephrine (10^–6^ mol·L^-1^). As previous study showed, the evaluation standard for endothelium-intact vessels is that the acetylcholine-induced relaxation achieves 80% or greater[9] and the same volume solvent alcohol was added as control group.

We also make natural hirsutine as a control to assess the relative potency of vasodilator effect of the analogue of hirsutine. The rings were pre-contracted with PE (10^–6^ mol·L^-1^), and then add to cumulative compound **1** or natural hirsutine with the concentration gradually increasing from 10^–11^ to 10^–4^ mol·L^-1^. Obtain the concentration-response curve and make comparison between these two groups.

#### Experimental Animals

There were seven groups in this experiment and each group has included at least six rats. Male Spontaneously Hypertensive Rats during 250–350 g were used. All animal care and experimental protocols complied with the Animal Management Rules of the Ministry of Health of the People’s Republic of China and approved by the local ethical committee of Fudan University. All surgery was performed under anesthesia of chloral hydrate or pentobarbital sodium, and all efforts were made to ameliorate animal suffering.

#### Induction of Intubation Technology

We use the Left Carotid Artery Intubation Technology to evaluating anti-hypertensive activity of compound **1.** Briefly, after anesthesia with chloral hydrate (20mg·kg^-1^), make the rat on supine position and implement endotracheal intubation. Separate the left carotid artery from the valgus and insert a polyethylene plastic pipe, which is filled with 0.1% heparin saline, into the artery pointing to the centripetal end. Then get the other end of the pipe throughout rat’s head back. Finally, suture the neck and head back incision and wait for the next day to let the rat awake. The rats should be maintained free for food or water during the next 12 hours before monitoring—blood pressure.

#### Drug Administration and blood pressure monitoring

On the next day, each rat was placed in a normal cage and we use the pipe out of their head back to connect with the blood pressure transducer via a tee interface, and transfer the digital information into computer by SMUP-PC biological signal processing system (Shanghai Jialong Instrument Factory, China). Compound **1** at the dose of 5, 10 and 20 (mg·kg^-1^) were respectively administered by tail vein injection in awake rats after record the basal blood pressure. Then do the same thing on the group of SHR. Then continuously observe and record the change of blood pressure and heart rate in 1 hour.

#### Compound 1-induced endothelium-dependent and-independent relaxations

The endothelium-intact and endothelium-denuded aortic ring were pre-contracted with PE (10^−6^ mol·L^-1^). Once get to the maximum isometric tension, continuous concentration–response curve of compound **1** (10^–11^ mol·L^-1^) were obtained. Make comparison between in absence and presence of endothelium groups.

#### Role of nitric oxide (NO) and guanylyl cyclase in compound 1-induced relaxation

Endothelium-intact aortic rings were pre-incubated for 15 min, respectively with L-NAME, a NO synthase inhibitor (10^–4^ mol·L^-1^)[10,11] and ODQ, a specific inhibitor of soluble guanylyl cyclase (10^–6^ mol·L^-1^)[12]. After that, aortic rings prior to contraction with PE (10^−6^ mol·L^-1^) to get to the plateau, then continuously add cumulative concentrations of compound **1** (10^-11^-10^-4^ mol·L^-1^) to the bath so as to obtain the concentration–response curve of compound **1**-induced relaxation. Compared with the control group to determine the response effect.

#### Role of muscarinic acetylcholine in compound 1-induced relaxation

To determine if muscarinic acetylcholine receptor play a role in compound **1**-induced vasodilation, add atropine (a acetylcholine receptor inhibition) at the dose of 10^–4^ mol·L^-1^ to the bath for 15 min before contracting the endothelium-intact rings with PE (10^−6^ mol·L^-1^), then use increasing concentrations of compound **1** (10^-11^-10^-4^ mol·L^-1^) to get the curve. Access the effect by comparing with the control group.

#### Effect of compound 1 on extracellular Ca^2+^-induced contraction activated by KCl

Calcium is an essential signaling molecule that controls vascular smooth muscle cells (VSMCs) contraction and nifedipine is a common calcium antagonist[13]. Aortic rings without endothelium were contracted by KCl (80 mM) to reveal whether the inhibition of extracellular Ca^2+^ influx had something with the compound **1**-induced relaxation. Then concentration–dependent curve of compound **1** was obtain by adding the cumulative compound (10^-11^-10^-4^ mol·L^-1^), then use nifedipine instead of compound **1** so as to get another curve. Finally, the compound **1** group was compared respectively with nifedipine group.

#### Role of the PE-induced sarcoplasmic reticulum calcium release

To clarify if the inhibition of intracellular Ca^2+^-release caused the compound **1**-induced vasodilation effect, firstly stimulate the release of sarcoplasmic reticulum calcium completely by contracting the rings in PSS with Ca^2+^ by PE as previously said until obtain the plateau. Then endothelium-denuded rings were washed by Ca^2+^-free PSS for three times and add compound **1** at different concentrations as follows (mol·L^-1^):10^–9^,10^–8^,10^–7^,10^–6^,10^–5^ and 10^–4^ which to be dependent groups. The compound was present during 15 min and at last, PE (10^−6^ mol·L^-1^) was added again to help the intracellular Ca^2+^ release. The maximal isometric tension induced by PE firstly (compound-free) was considered as 100%.

#### Role of potassium channels in compound 1-induced relaxation

In order to know the role of K^+^ channels on compound **1**-induced relaxation, arterial rings whose endothelium-denuded incubated in advance using the K^+^ channel blockers, TEA (5×10^−6^ mol·L^-1^), glibenclamide (10^−5^ mol·L^-1^) for 15 min before stimulated by PE (10^−6^ mol·L^-1^), and then the compound was added into the bath continuously from 10^–11^ to 10^–4^ mol·L^-1^.

### Data analysis

Firstly, we calculated the contraction value of each compound at each time point by EXCEL, using the following formula:

$$\text{Contraction value}(\text{y-axis})=\frac{\text{each compound tension-baseline tension}}{\text{Maximum tension -baseline tension}}$$

And then do the analysis of IC 50 with one-way ANOVA as well as Turkey's multiple comparison test. The data of curves would be modified by a nonlinear regression method (GraphPad Prism). Results were presented as means±standard error of the mean (S.E.M.). What’s more, we do analysis of P value with two-way ANOVA from GraphPad Prism. The data of the two groups would be compared with a repeated measures ANAVO method.

## Results

### Toxicity test by MTT and LDH Elisa kit

**Fig 2:**
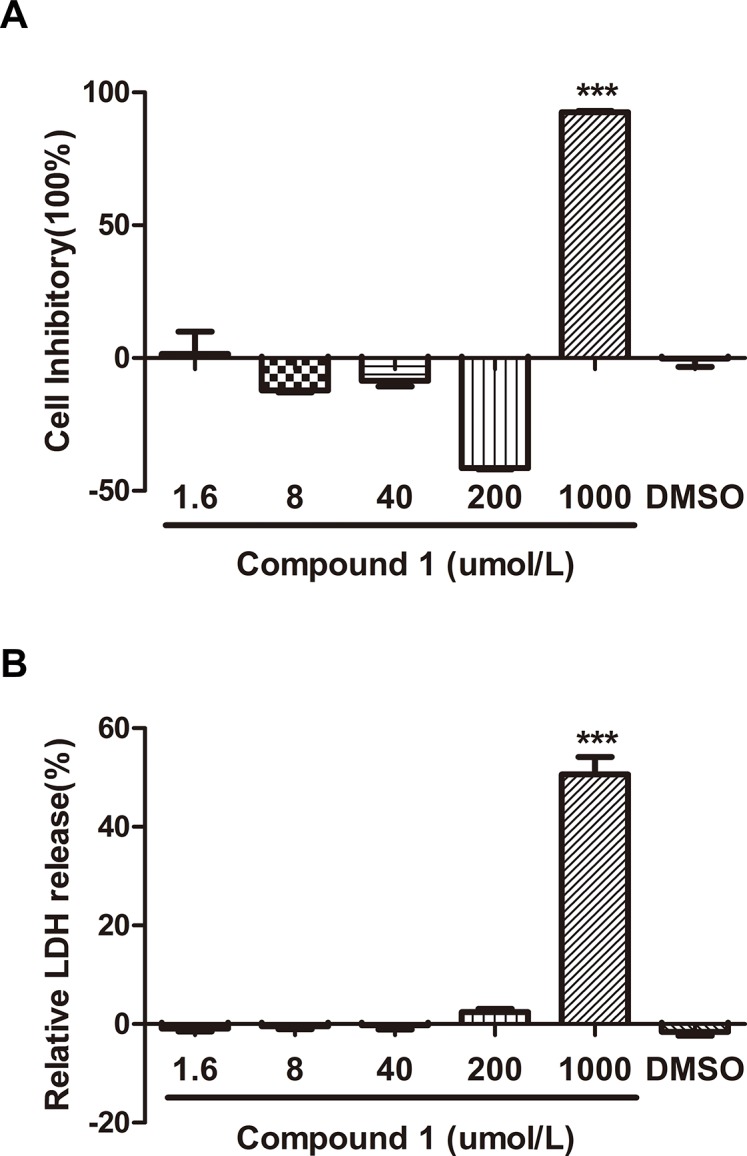
Toxicity test by MTT and LDH Elisa kit. A. The MTT assay. The toxicity was accessed by measuring cell death using the MTT assay. B. LDH release assay. For each group was standardized with the respective control and results were as the mean ± S.E.M., n = 6, ***P<0.001, compared with the DMSO group.

Compound **1** had no toxicity on cell viability during the normal dosage range, as accessed by MTT assay, compared with the normal control group. When VSMCs were treated with compound **1** (1.6,8,40,200,1000μΜ) for 12h, only the 1mM group had toxicity on the cells respectively compared with the control group (**Fig 2A)**.

Lactate dehydrogenase leakage (LDH) evaluated also has no more higher than in the control group during the normal dosage range and compound **1** had indeed no effect on cell death, which was consistent with the result observed with the MTT assay **(Fig 2B)**.

### Effect of vasodilation and anti-hypertension

**Fig 3:**
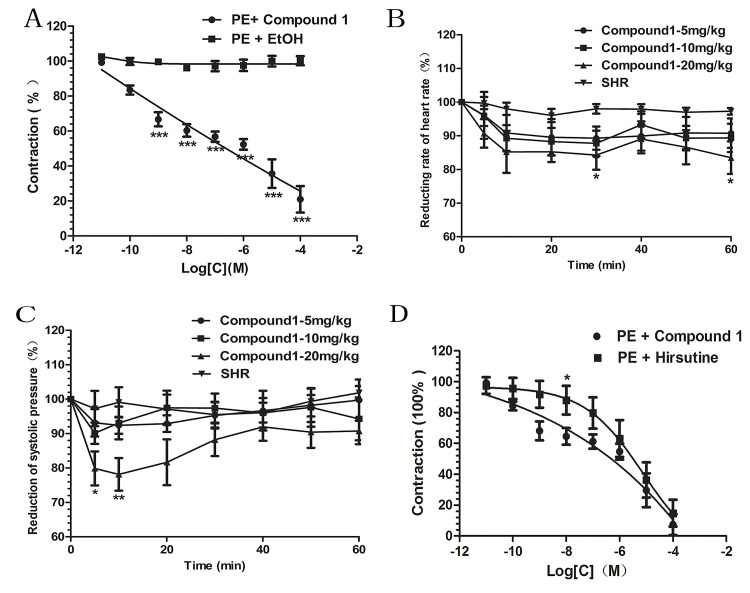
Effect of vasodilation and anti-hypertension. A. Cumulative concentration of compound **1**-induced relaxation precontracted with 1 μM PE in rat aortic rings with functionally intact endothelium. Means ± S.E.M, n = 6 rings,***P<0.001vs Ethanol control group. B. The reduction rate of systolic blood pressure (SBP). C. The reduction rate of heart rate. Results are presented as mean ± S.E.M., n = 6, *P<0.05, **p<0.01vs SHR group. D. The different effect between compound **1** and natural hirsutine relaxation. Results are presented as mean ± S.E.M., n = 6, *P<0.05 compared with compound 1 group.

Compound **1** could diastole aortic rings with endothelium after stimulated by PE (10^−6^ mol·L^-1^) in a dose-dependent manner as well as the IC_50_ (Concentration necessary to reduce maximal phenylephrine induced contraction by 50%) was 1.129×10^–9^±0.5025 **(Fig 3A)**.

In vivo study, systolic blood pressure (SBP) and heart rate (HR) were recorded for 1h and baseline parameter before injecting compound **1** (5,10,20 mg·kg^-1^) were considered with 100% of activity. There is a significant reduction in SBP and HR during the first 0.5h after administrating 20mg/kg compound **1** and the anti-hypertensive effect of compound **1** was slowly and continuously get to the basal line **(Fig 3B and 3C)**.

In further study, we found that the vasodilation effect of compound **1** could not be significant differences than that of natural hirsutine, only at the concentration of 10^-8^M could show the obvious differences (P<0.05). That is to say, our novel compound has the similar great vasodilation effect compared with the natural one. Meanwhile, it is easier to synthesize compound **1 (Fig 3D)**.

### Compound 1-induced endothelium-dependent and-independent relaxations

**Fig 4:**
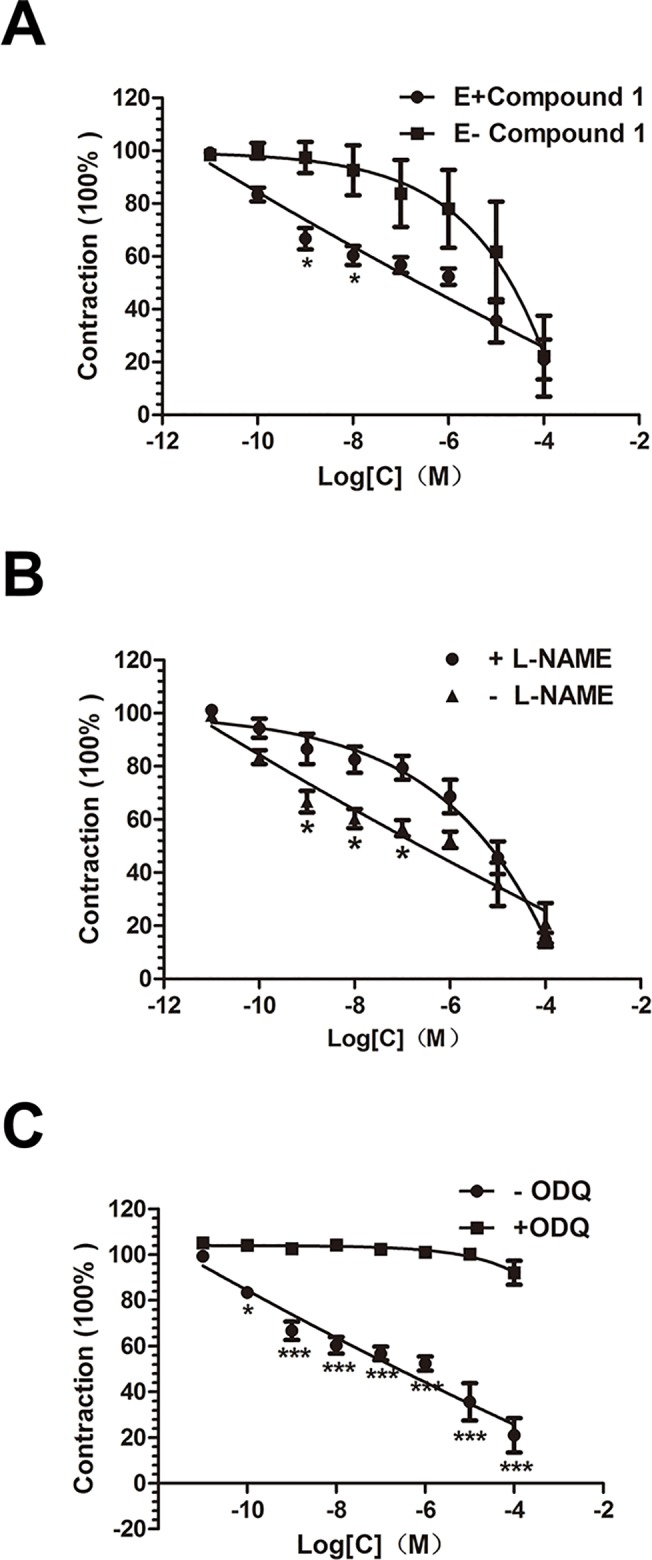
Compound 1-induced endothelium-dependent and-independent relaxations. a. The differences between compound **1**-induced endothelium-dependent and—independent relaxation. b. Effects of L-NAME (1uM) on compound **1**-induced relaxation in endothelium-denuded aortic rings. c. Effects of ODQ (1uM) on compound **1**-induced relaxation in endothelium-denuded aortic rings. Results are presented as mean ± S.E.M., n = 6, *P<0.05,**p<0.01,***P<0.001 compared with compound-only group.

The vasodilation effect induced by compound **1**, with PE (10^−6^ mol·L^-1^) pre-contracted, was significantly impaired when endothelium cells were removed physically according to the comparison between E+ and E- groups **(Fig 4A)** which showed that the vasodilation was in partially endothelium-dependent way.

### Role of nitric oxide and guanylyl cyclase in compound 1-induced relaxation

For further study the eNO synthase had been confirmed to be involved in this vasodilation process **(Fig 4B)**. What’s more, the use of ODQ can almost completely inhibit the compound **1**-induced relaxation **(Fig 4C)**. So NO and sGC would have important effect on the issue how compound **1** played diastole role through endothelium-dependent manner.

### Role of muscarinic acetylcholine in compound 1-induced relaxation

**Fig 5:**
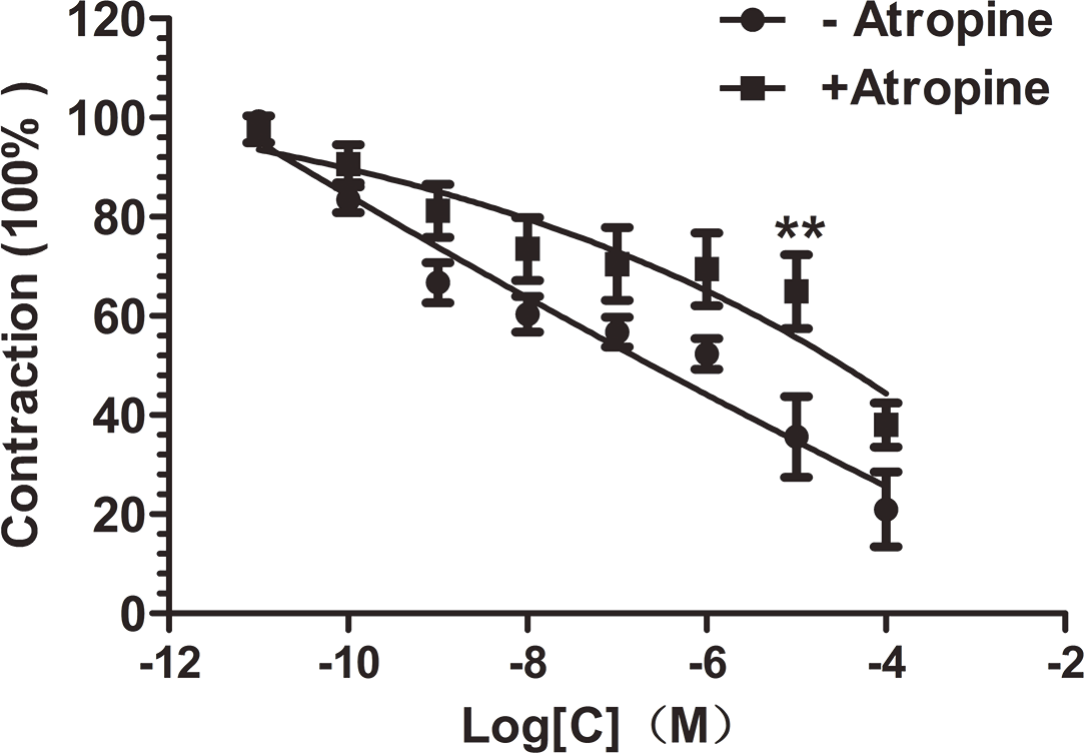
Role of Atropine (1 × 10^–6^ mol·L^-1^) in the vasodilation induced by compound 1. Use the endothelium-denuded aortic rings. Data are showed as mean ± S.E.M., n = 6, *P<0.05

Through this experiment the M-R function in compound **1**-induced relaxation can be excluded because only at the concentration of 10^–5^ mol·L^-1^ compound **1** did the Atropine at presence or absence groups have significant differences **(Fig 5)**, and this result had no concentration-response trend.

### Effect of compound 1 on extracellular Ca^2+^-induced contraction activated by KCl

**Fig 6:**
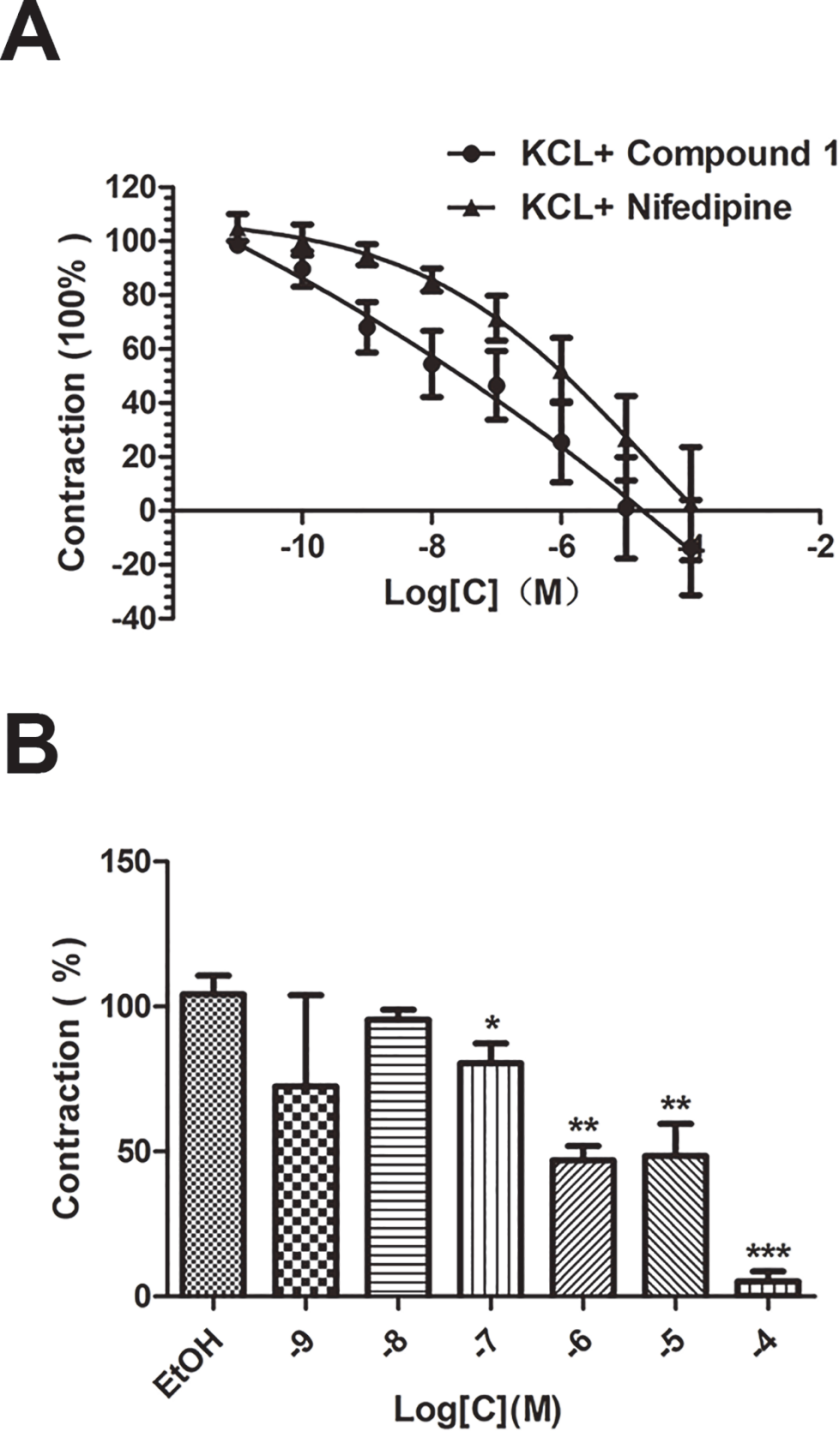
Role of calcium channel in compound 1-induced relaxation. A. Comparison of compound **1** and nifedipine on KCl-activated (80mM) contraction in the endothelium-denuded rings. B. Role of the PE-induced sarcoplasmic reticulum calcium release. Data are showed as mean ± S.E.M., n = 6, *P<0.05,** P<0.01,*** P<0.001.

When respectively compared the compound **1** group with the nifedipine group, we can find that there is no distinctly differences between the two groups but the relaxation trend of compound **1** is as same as nifedipine **(Fig 6A)**, which indicated that compound **1** relaxed vessels partially through inhibiting extracellular Ca^2+^ influx.

### Role of the PE-induced sarcoplasmic reticulum calcium release

In the Ca^2+^-free PSS, incubated with compound **1** (10^–9^,10^–8^,10^–7^,10^–6^,10^–5^ and 10^–4^ mol·L^-1^) for 15 min after completely release the intracellular Ca^2+^ and from the concentration of 10^–7^ mol·L^-1^ that compound **1** could evidently attenuate PE (10^−6^ mol·L^-1^) induced contraction **(Fig 6B)**, showed that the compound also cause an inhibition on the sarcoplasmic reticulum calcium release.

### Role of potassium channel in compound 1-induced relaxation

**Fig 7:**
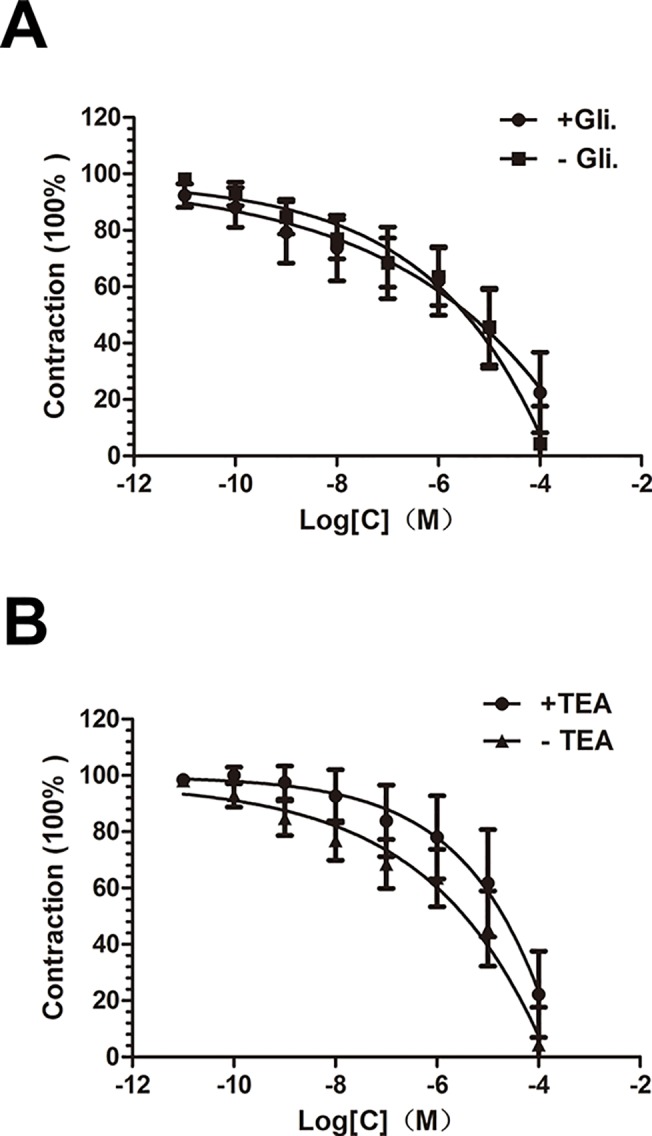
Role of potassium channel in compound 1-induced relaxation. A. Role of glibenclamide (10^**−5**^ mol·L^**-1**^) on no-endothelium aortic rings pre-stimulated by PE(10^**–6**^ mol·L^**-1**^) in the vasodilation induced by compound **1**. B. Effect of TEA (5x10^**−6**^ mol·L-1) on compound **1** vasodilation. Data are showed as mean ± S.E.M., n = 6.

Both of the K^+^ channel blockers, glibenclamide (10^−5^ mol·L^-1^) or TEA (5x10^−6^ mol·L^-1^), had no obviously effect on inhibiting compound **1**-induced relaxation in endothelium-denuded rings pre-stimulated by PE (10^–6^ mol·L^-1^) **(Fig 7A and 7B)**.

## Discussion

Previous studies show that hirsutine can relax the aortic vessels against phenylephrine-induced contraction in a concentration-dependent way[3]. To develop more potent compounds, an analogue of hirsutine, compound **1** was designed basing on the structure of hirsutine. The pharmacological studies of compound **1** show that it can vasodilator thoracic aorta at a very low concentration (IC_50_ is 10^–9^ mol·L^-1^), and the animal studies exhibit that compound **1** can evoke a quick and significant effect on reducing basal SBP and HR (P<0.05) by intravenously administration of compound **1** in SHR, which is consistent with the vasodilation results and further demonstrated the anti-hypertensive effect of this compound.

Differing from nifedipine that could induce blood pressure reduction accompanied with a sustained elevation of the HR[14], compound **1** can decreases both BP and HR what would be safer for the therapy of cardiovascular diseases. Meanwhile, we find that compound **1** exhibits extremely low toxicity under effective dose, which indicates that compound **1** will be an ideal and safe medicine candidate.

As far as we are concerned, the putative mechanisms eventually involved in the blood pressure changes and in vasodilation of hirsutine have been studied in many other researches before. In general, taking the mechanisms involved in hirsutine’s anti-hypertension effect into consideration[15], we assumed that both endothelium dependent and independent ways play a role on compound **1**’s vasodilatation effects. So we designed those experiments related to NO/cGMP pathway, muscarinic acetylcholine function, Ca^2+^ channel as well as K^+^ channel and identify whether they can have effects on the vasodilation of compound **1**.

From the results that compound **1** impacted PE-induced contraction on endothelium-intact and-denuded aortic rings, we can see significant differences which indicate endothelial integrity involved in the vasorelaxation caused by compound **1**. Furthermore, we find that L-NAME, an inhibitor of eNOS, can also block the effect of compound **1**. As the cardiovascular effect of nitric oxide is mainly mediated by activation of soluble guanylate cyclase (sGC), in next step the aorta from SD rats was pre-treated by ODQ, an inhibitor of sGC, and we observed that it could distinctly weaken the response triggered by compound **1** in endothelium-intact vessels, suggesting that cyclic nucleotide-cGMP might be involved in the compound **1**-mediated vasodilation. Therefore, these data demonstrated that compound **1** dilates vessels in endothelium-dependent manner which particularly related to NO release from endothelial resulted from cGMP activation, then bring about vasodilatation, that is to say, the NO/cyclic GMP pathway is involved in the relaxation progress.

And we can obtain the conclusion from the next experiment that the muscarinic acetylcholine effect in compound **1**-induced relaxation mechanism can be excluded since Atropine at presence or absence groups have no significant differences and we cannot see the concentration-response trend.

Then as we all know, high K^+^-induced (80mM) contraction in endothelium-denuded aortic rings is due to cell membrane depolarization and activation of the voltage-dependent Ca^2+^ channel, especially for L-type Ca^2+^ channel which could promote Ca^2+^ influx[16,17]. According to our present study, both compound **1** and nifedipine can inhibit high K^+^-induced contraction in a concentration-dependent manner. On the other hand, compound **1** also has significant effect on phenylephrine-induced contraction in a dose-dependent manner, which mostly related to receptor-operated Ca^2+^ channel. The inositol trisphosphate (IP_3_) can activates the IP_3_ receptor (IP_3_R) to release stored calcium (Ca^2+^) from sarcoplasmic reticulum[18]. All of these results preliminarily reveal that the mechanism of compound **1**-induced dilation in vascular smooth muscle tissues includes both inhibiting extracellular Ca^2+^ entry by L-type Ca^2+^ channel in depolarized tissues and blocking the intracellular Ca^2+^ release from sarcoplasmic reticulum through a possible IP_3_ signaling pathway[19]. Another important ion channel, K^+^ channel also has close ties to vessel relaxation. There are two main K^+^ channels we studied in this content: the non-specific ATP-sensitive K^+^ channel and the Ca^2+^-activated K^+^ channel[20]. Pre-treatment with glibenclamide or TEA cannot change the concentration-dependent curve relaxed by compound **1**. So neither of these two K^+^ channels has significant effect on compound **1**-induced dilation which suggests that opening K^+^ channels is not involved in the mechanism of action of compound **1**.

Further, some possible confounding factors and possible study limitations, such as evaluating 24-hour urinary sodium and potassium excretion, were not related to some related mechanisms of action. Firstly, we can see that the potassium channel has no effect on compound **1**’s vasodilation and then those tests related to potassium channel are not very necessary to do. What’s more, we have no enough idea about compound **1** so far and just want to get initial understanding of it. As we know, the icon channels may play the roles because of activating some proteases, the progress of which is transient and in a short time so that we cannot get the significant differences through those long-term study. Considering all of these factors, we didn’t design the long-term experiments in our current study.

In conclusion, our results suggests that compound **1** can induce vasodilation in rat aortic rings and reduction in SBP and HR which mediated by both endothelium-dependent and-independent manners, and NO/cyclic GMP pathway can be involved in endothelium-dependent manner. Meanwhile the mechanism on compound **1**-induced vasodilatation probably also related to blockade of Ca^2+^ influx through L-type Ca^2+^ channels and inhibition of intracellular Ca^2+^ release.
